# Dietary Polyphenols as Therapeutic Intervention for Alzheimer’s Disease: A Mechanistic Insight

**DOI:** 10.3390/antiox11030554

**Published:** 2022-03-15

**Authors:** Syed Nasir Abbas Bukhari

**Affiliations:** Department of Pharmaceutical Chemistry, College of Pharmacy, Jouf University, Aljouf 2014, Saudi Arabia; sbukhari@ju.edu.sa or snabphd@yahoo.com; Tel.: +966-565-738-896

**Keywords:** amyloid, like curcumin, quercetin, homeostasis, metal chelation

## Abstract

Dietary polyphenols encompass a diverse range of secondary metabolites found in nature, such as fruits, vegetables, herbal teas, wine, and cocoa products, etc. Structurally, they are either derivatives or isomers of phenol acid, isoflavonoids and possess hidden health promoting characteristics, such as antioxidative, anti-aging, anti-cancerous and many more. The use of such polyphenols in combating the neuropathological war raging in this generation is currently a hotly debated topic. Lately, Alzheimer’s disease (AD) is emerging as the most common neuropathological disease, destroying the livelihoods of millions in one way or another. Any therapeutic intervention to curtail its advancement in the generation to come has been in vain to date. Using dietary polyphenols to construct the barricade around it is going to be an effective strategy, taking into account their hidden potential to counter multifactorial events taking place under such pathology. Besides their strong antioxidant properties, naturally occurring polyphenols are reported to have neuroprotective effects by modulating the Aβ biogenesis pathway in Alzheimer’s disease. Thus, in this review, I am focusing on unlocking the hidden secrets of dietary polyphenols and their mechanistic advantages to fight the war with AD and related pathology.

## 1. Introduction

Alzheimer’s disease falls under the category of chronic neurodegenerative diseases and is considered the most prevalent cause of dementia worldwide. Statistical data suggest that approximately 40 million people are suffering from age related dementia globally, and this number is supposed to be doubled until approximately 2050 [1]. Alzheimer’s disease has a well-documented neuropathology, which is characterized by the formation of extracellular amyloid plaques and intracellular Tau neurofibrillary tangles (NFTs) in the medial temporal lobe and neocortical structures in the brain [2]. The amyloid, also termed senile plaques (SPs), is mainly composed of proteinaceous components called Aβ peptides, which are formed by the cleavage of the large amyloid precursor protein (APP) [3]. APP is sequentially cleaved by two enzymes, γ-secretase and β-secretase (BACE1), majorly producing Aβ38, Aβ40, and Aβ42 as the most common variants. According to the “amyloid hypothesis”, accumulation of Aβ in the brain triggers a cascade that results in the formation of neurofibrillary tangles via tau protein hyperphosphorylation and also initiates multifactorial biochemical responses ranging from local inflammation, cytokine release, oxidative stress and excitotoxicity [4,5,6]. Consequently, progressive structural changes in surrounding neuronal cells that are characterized by synapse loss, imbalance between neurotransmitters (e.g., acetylcholine, dopamine), and neuron death ultimately lead to cognitive failure in AD patients [3,7,8,9]. Alzheimer’s disease has a median duration of 10 years. As the disease progresses, patients report difficulties in communication, executive function, recognition of direction, learning capabilities, and cognitive thinking [9,10]. Various parameters such as genetic, epigenetic, environmental factors, lifestyle and comorbidities also contribute to the progression of this complex neurodegenerative disorder.

In the last decade, tremendous efforts have been made in clinical research to understand the pathogenesis of Alzheimer’s disease as well as in the development of novel Alzheimer’s disease therapeutics. However, a few clinically relevant medicines such as levodopa and tetrabenazine are available to subside the symptoms of Alzheimer’s disease and delay the progression of this devastating disease [11,12,13,14]. Alzheimer’s disease is treated with acetylcholinesterase inhibitors (AChEI) and the *N*-methyl-d-aspartate receptor (NMDAR) antagonist memantine. Side effects can occur even with reasonably safe pharmaceuticals such as AChEIs and memantine. This can lead to a decrease in quality of life, incorrect prescription cascades, or even death, thus it is crucial to be informed of potential side effects [15]. Due to limited medical therapeutics and harsh side effects, there is an immediate requirement for alternative, preventive, and dietary approaches that can restrain the manifestation and progression of Alzheimer’s disease. Taking this into consideration, several studies have been carried out to elucidate the beneficial aspect of diet in the prevention of disease progression [16,17,18]. The growing bodies of evidence suggest that the high intake of vitamins such as vitamin C, E, flavonoids, PUFA (unsaturated fatty acid), folate, polyphenols, and other dietary restrictions has been associated with a reduced risk of Alzheimer’s disease [19,20,21,22]. In recent years, the Mediterranean diet has been receiving great attention not only for lowering the risk of neurodegenerative disorders, but also because its intake is associated with a reduced manifestation of cardiovascular disease and a variety of cancers [22]. Moreover, The Washington Heights-Inwood Columbia Aging Project also provides clues to the positive association of the Mediterranean diet with a lower risk of Alzheimer’s disease and related cognitive deficiencies [23]. The Mediterranean diet is a plant-enriched diet with a high content of phytochemicals called polyphenols. Polyphenols are naturally occurring substances present in plants, fruits, and vegetables and are known to have neuroprotective effects. There are many important dietary sources of polyphenols, including fruits (apple, berries, cocoa), vegetables, herbs, grains, red wine, nuts, tea, onions, and seeds [22]. It has been reported that dietary polyphenols prevent the pathological manifestations of Alzheimer’s disease due to their ability to cross the blood-brain barrier [24,25]. The neuroprotective effects exhibited by dietary polyphenols may be because of their antioxidant and anti-inflammatory properties, but circumstantial evidence also suggests that their beneficial role is attributed to novel therapeutic routes and targets by modulating intracellular signaling pathways, gene expression, and enzyme activity in neurodegenerative diseases [26,27,28,29]. This review critically outlines the therapeutic role of dietary polyphenols in ameliorating the progression of Alzheimer’s disease by targeting the disease’s neuro-pathophysiology, based on the most recent scientific literature.

## 2. Molecular Mechanisms and Pathology of Alzheimer’s Disease

**Figure 1:**
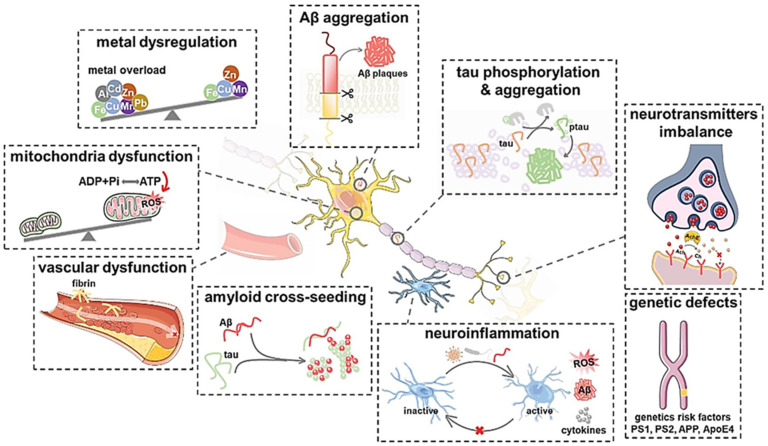
Different plausible Alzheimer’s disease mechanisms of Aβ aggregation, tau phosphorylation and aggregation, neurotransmitters imbalance, genetic defects, neuroinflammation, amyloid cross-seeding, vascular dysfunction, mitochondria dysfunction, and metal dysregulation [31]. “Reprinted from [31] “A mechanistic survey of Alzheimer’s disease” Biophysical Chemistry 2022, 281, 106735, (2022), with permission from Elsevier”.

**Figure 2:**
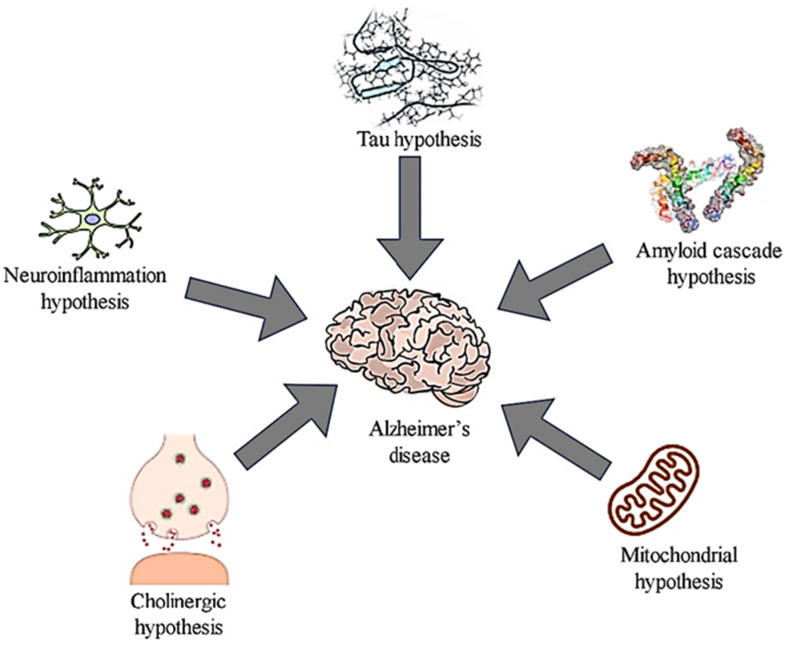
Proposed Alzheimer’s disease Hypothesis and the mechanisms involved [32]. “Reprinted from “Nanocarrier mediated drug delivery as an impeccable therapeutic approach against Alzheimer’s disease. Journal of Controlled Release 2022, 343, 528–550, Taliyan, R.; Kakoty, V.; Sarathlal, K.C.; Kharavtekar, S.S.; Karennanavar, C.R.; Choudhary, Y.K.; Singhvi, G.; Riadi, Y.; Dubey, S.K.; Kesharwani, P. Copyright (2022), with permission from Elsevier”.

Alzheimer’s disease is defined pathologically by gradual synaptic and neuronal degeneration and the appearance of diagnostic amyloid plaques consisting of fibril β-amyloid peptide aggregates and neurofibrillary tangles comprising hyperphosphorylated tau protein filaments. While plaques and tangles were first thought to be the primary mediators of neurotoxicity in Alzheimer’s disease, new research has established the importance of soluble-amyloid oligomers and tau molecules [30], (Figure 1 and Figure 2).

## 3. Therapeutic Strategies Based on Symptoms of Alzheimer’s Disease

Although Alzheimer’s disease is recognized as a public health problem, only two classes of drugs have been approved to treat it: cholinesterase enzyme inhibitors and N-methyl d-aspartate (NMDA) antagonists [2]. During the progression of Alzheimer’s disease, a reduction in biosynthesis of acetylcholine is observed. Acetylcholinesterase inhibitors (AChEIs) specifically block the activity of cholinesterase enzymes, which results in an increase in ACh levels in the synaptic cleft. As a result, blocking acetylcholinesterase (AChE) activity is one of the most common ways to fight Alzheimer’s disease symptoms.

The first approved cholinesterase inhibitor drug was Tacrine (tetrahydroaminoacridine) for the treatment of Alzheimer’s disease symptoms, which increases ACh in neurons, but during several trials, it was found to have hepatotoxic effects [2,33]. Nowadays, several AChEIs are available in the market, such as donepezil, rivastigmine, and galantamine, and are utilized popularly to treat symptoms of Alzheimer’s disease [11,12,13,14].

Another strategy that may help in the treatment of Alzheimer’s disease is using the antagonist of NMDA. The NMDA receptor plays a dominant role in the progression of Alzheimer’s disease. Upon stimulation of the NMDA receptor, a Ca^2+^ influx activates a signaling pathway that triggers the activation of genes involved in the formation of long-term potentiation (LTP). Overstimulation of NMDARs results in an abnormal increase in the Ca^2+^ influx which causes excitotoxicity damage, synaptic dysfunction, apoptotic cell death, and cognitive decline [34,35]. A wide range of NMDAR antagonists, such as Memantine and RL-208, have been developed to treat moderate to severe symptoms of Alzheimer’s disease [36,37,38]. These drugs are used to relieve patients of Alzheimer’s disease symptoms, but highly potent, selective, and effective approaches are immediately required to treat Alzheimer’s disease and other related dementia disorders.

## 4. Therapeutic Strategies Based on Targeting Different Alzheimer’s Marker

### 4.1. Metal Chelation Approach

The metal chelation approach is an emerging area that can be effective in managing Alzheimer’s disease. Support for this can be inferred from the studies in which they have found that upon undergoing association with metal ions such as Cu^2+^, Zn^2+^ and Fe^2+^, Aβ aggregation propensity increases with a subsequent increase in oxidative stress response [39,40]. Already, metal chelation has been identified as one of the mechanisms associated with the protective response of a few polyphenols [28]. Although, metal chelation’s effect on the progression of Alzheimer’s pathology is at its nascent stages, the level of metal ions has a high variability among different age groups. 

### 4.2. Amyloid Fiber Disruption Strategy

The most interesting features that define Alzheimer’s disease are the formation of Aβ fiber and hyperphosphorylation of τau protein [4,41]. Directly targeting the process connected with their biogenesis is an interesting proposition to look into. Interference in this toxic program can be achieved by molecular entities exhibiting the property of adduct formation with Aβ which decreases their aggregation propensity. Furthermore, converting the soluble protein into an insoluble form upon adduct formation has been found to be associated with a decrease in their toxicity response under Alzheimer’s conditions [42,43].

### 4.3. Antioxidant Approach

It has been seen that oxidative stress is a prime contributor to the progression of Alzheimer’s’ pathology [44]. Approaches that decrease the oxidative burden are effective in the amelioration of Alzheimer’s disease [45,46]. In that direction, potential antioxidants can be explored, which can have an effectivity in managing Alzheimer’s disease.

### 4.4. Targeting Protein Homeostasis

The formation of Aβ aggregate results from an imbalance in the protein homeostasis mechanism inside cells. An unfolded protein response (UPR) is one such response that gets activated in response to any disordered protein. Strategies employing the abrupt activation of UPR are effective in the amelioration of Alzheimer’s disease pathology [47]. Moreover, unstructured or defective proteins are subjected to the ubiquitin proteasomal system (UPS) mediated degradation process. Defective UPS responses are associated with an exacerbation of Alzheimer’s pathological responses [48,49]. Therefore, maintaining the homeostasis of UPS can be an effective approach for fighting the Alzheimer’s disease pathological response.

### 4.5. Anti-Inflammatory Approach for Alzheimer’s Disease

Ageing-activated inflammatory cascades are thought to play an important role in modulating the course of ageing associated with neurodegenerative disorders [50]. The role of inflammatory cascades in amplifying Alzheimer’s pathology have been established in the past [51]. Hence, targeting these inflammatory events is an effective approach used for therapeutic advantage in Alzheimer’s disease.

### 4.6. Dietary Approaches for Alzheimer’s Disease

Several studies have suggested the therapeutic role of diet and nutrition in the progression and management of Alzheimer’s disease. The Mediterranean diet has gained popularity among the population due to its association with low morbidity and mortality linked to neurodegenerative diseases [16,52]. The Mediterranean diet is known to be enriched in polyphenols as it is characterized by a relatively high consumption of fruits and vegetables that are rich sources of polyphenols. Scarmeas et al., 2007 [53], studied whether a high intake of polyphenols in the form of fruits and vegetables is linked with a reduced risk of Alzheimer’s disease. However, there are several studies showing that the use of polyphenols reduces the occurrence of inflammation, a condition closely connected with a number of chronic diseases and health conditions [26,54].

## 5. Basics of Dietary Polyphenols

**Figure 3:**
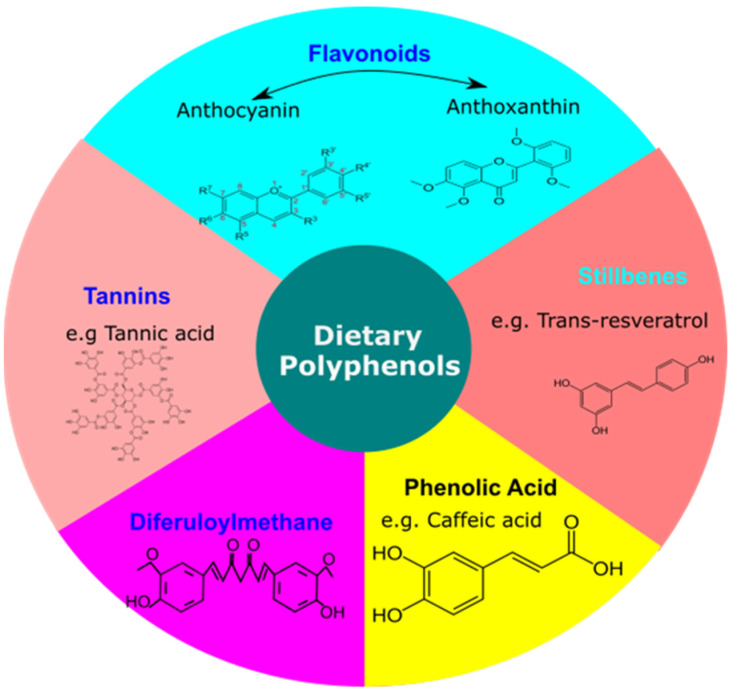
Classification of Dietary Polyphenols. Basic classification includes flavonoids (Anthocyanin, Anthoxanthin), stilbenes (Trans-resveratrol), phenolic acid (caffeic acid), diferuloylmethane, tannins (tannic acid).

Dietary polyphenols constitute the major class of antioxidants consumed on a daily basis across the globe, and their use for human health has received tremendous attention. Polyphenols play an important role in the prevention of a variety of diseases, particularly cardiovascular disease, cancer, diabetes, and neurodegenerative diseases, according to a growing body of evidence [22,55]. Polyphenols are regarded as strong antioxidants that work against oxidative stress caused by the excess accumulation of free radicals. In recent years, certain evidence has also indicated that, beyond their popular antioxidant activity, they may succeed by modulating cell signaling pathways [56,57]. Structurally, polyphenols are a group of natural compounds that consist of phenolic rings. They are formally characterized by the presence of an aromatic ring in their structure, having different levels of hydroxyl moiety attached to them. Structurally, approximately 8000 different structural forms of these polyphenolic compounds present in various food sources have been identified to date. Fruits and beverages represent the predominant source of dietary polyphenols, followed by vegetables, cereals and dry legumes. It has been reported that different polyphenol groups may have a different stability, bioavailability and physiological functions [58]. These polyphenolic compounds are further classified as diferuloylmethane, stilbenes, flavonoids, phenolic acids and tannins (Figure 3). It has been reported that different polyphenol groups may have different stability, bioavailability and physiological functions. Phenolic acid constitutes one third of our dietary intake and remaining two thirds are contributed by flavonoids. A major class among phenolic acids consists of hydroxycinnamic acids such as caffeic acid, which is present in an esterified form with chlorogenic acid. They are found to have anti-carcinogenic properties through their role in interfering with the nitrosylation process in the biological system. On the other hand, flavonoids are classified into anthocyanin (colored in nature & mainly present in colored fruits and vegetables) and canthaxanthin (colorless further classified under flavones, flavanols, flavans, isoflavones). Stilbenes (e.g., trans-resveratrol) have a characteristic 1,2-diphenylethylene group and are present in their monomeric and oligomeric forms in nature. Tannin is the water-soluble form of a dietary polyphenol having a molecular weight in the range of 500 to 3000. Diferuloylmethane has aromatic rings substituted with hydroxyls. They are further linked by carbonyl groups containing aliphatic chains. These polyphenols are readily available in fruits (apple, grapes, berries), vegetable herbs, grains, onions, red wine, etc.

The neuroprotective as well as antioxidant properties of the dietary polyphenols are associated with the modulation of Alzheimer’s disease related pathologies, suggesting a new approach for research and clinical interventions to treat neurodegenerative diseases.

## 6. Polyphenols’ Role in Oxidative Stress and Alzheimer’s Disease

**Figure 4:**
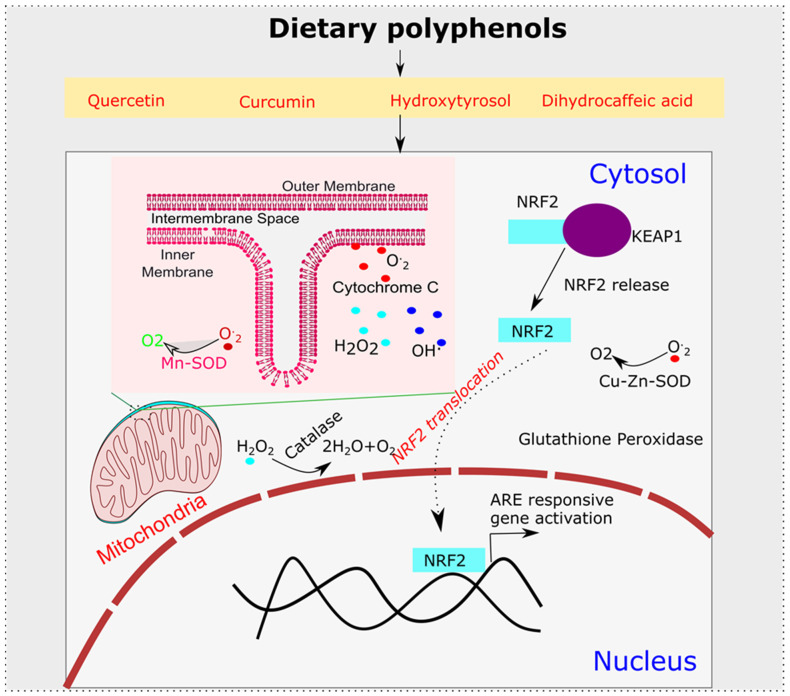
Dietary Polyphenols’ Anti-Oxidative Mechanism. Mostly dietary polyphenols’ basic mechanism of action involves their role as antioxidant which can be achieved by modulating NRF2-KEAP1 antioxidant response pathway or through modulation of antioxidant enzyme, such as SOD (Superoxide Dismutase), Catalase and Glutathione peroxidase.

Alzheimer’s disease is the most common cause of disability in adults over 65 years of age. The role of oxidative stress in the initiation or enhancement of this pathological cascade seems to be an important factor. In general, oxidative stress is a state of imbalance between oxidative and anti-oxidative systems in favor of oxidative machinery. A major source of oxidative stress is the conversion of oxygen to a superoxide radical, which further, upon the addition of one more electron, gets converted to hydrogen peroxide. Hydrogen peroxide with further oxidation produces hydroxyl radicals with a high oxidative potential. They are found to oxidize proteins, lipids and nucleic acids to a great extent, thus resulting in an alteration in their biological functioning. In a normal cell, 98% of oxygen is consumed by the electron transport chain and the remaining 2% gets converted to superoxide which has been taken care of by the cellular anti-oxidative defense system. To prevent mitochondria from oxidative damage, our system has developed various mechanisms. Mn-SOD, which is found in the mitochondrial matrix, aids in the reduction of superoxide produced during respiration [59,60]. The presence of Cu-Zn-SOD in the cytosolic space of cells further mitigates the effects of superoxide and hydrogen peroxide radicals [61]. In the mitochondrial membrane itself, Cytochrome C also helps to regenerate molecular oxygen from superoxide radicals [62]. Furthermore, glutathione peroxidase and catalase are two additional lines of defense built by the cellular system to combat oxidative stress [63]. With ageing, the level of oxidative stress increases, which further changes the molecular architecture of the cellular environment. This increase in oxidative stress is a causative factor for the development of Alzheimer’s disease, which is evident from the high toxic responses seen in Aβ-copper as compared to Aβ [64]. Copper is a mediator of hydroxyl radical mediated oxidative stress. Therefore, oxidative stress plays a significant role in the development of the pathology of Alzheimer’s disease. In addition, an increased level of zinc in cognition associated regions, such as the neocortex, amygdala and hippocampus, adds further evidence for metal catalyzed oxidative reactions and their role in Alzheimer’s pathologies [65,66]. Therefore, any agent or molecular entity that can bring an equilibrium between these antioxidative defense mechanisms is going to be important for countering Alzheimer’s disease. Dietary polyphenols perfectly fit the above criteria. It has been shown that polyphenols exert antioxidative function either directly through the scavenging of free radicals or by increasing the capacity of the endogenous defense system. Among polyphenols, dihydrocaffeic acid is found to have a free radical scavenging capability [66]. Glutathione peroxidase and superoxide dismutase were found to be induced after the application of curcumin and quercetin [67,68,69,70]. Hydroxytyrosol was also found to have an influence on the activity of catalase and superoxide dismutase (SOD) [71,72]. Such antioxidative function is further orchestrated by the modulation of the KEAP-ARE axis, which is an important counter of the oxidative and xenobiotic stress. Epigallocatechin and quercetin were found to influence this ARE axis to decrease the oxidative burden [73,74] (Figure 4). Furthermore, curcumin modulation of the ARE axis through NRF2 modulation was found to increase the Glutathione S-Transferase P1 functionality [75].

## 7. Selected Polyphenols Used in Treatment against Alzheimer’s Disease

### 7.1. Mechanistic Involvement of Curcumin in Alzheimer’s Disease

Among dietary polyphenols, curcumin’s usage on the south Asian continent has been documented for centuries. Its anti-inflammatory, antibacterial, and anticarcinogenic effects have resulted in its widespread use in Ayurvedic practices as well. In natural extracts isolated from turmeric rhizome, various forms of curcumin, collectively called curcuminoids, such as curcumin (curcumin-I), desmethoxycurcumin (curcumin-II), bisdemethoxycurcumin (curcumin-III) [76], have been found. Their collective action is responsible for the protective effect seen in cases of various pathologies. In the case of Alzheimer’s disease, a population-based study conducted to compare the incidences of Alzheimer’s disease has identified 4.4-fold higher incidences of Alzheimer’s disease in the US population in the 70–79 age group when compared to the rate of incidences in India. The reason for this was inferred to be common dietary usage of turmeric which contains curcumin as the principal constituent [77,78]. Several scientific studies have already identified curcumin as interfering with Aβ biogenesis and also aiding in the clearance of its deposits, thereby preventing the formation of Aβ aggregates [21,27,79]. Owing to the role of oxidation in the development of Alzheimer’s disease, curcumin’s associated antioxidative effect might play a key role in beneficial effects seen after curcumin usage in Alzheimer’s disease. Such effects of curcumin on a molecular level are coordinated by NF-κβ, which is a prime transcription factor that is responsible for the orchestration of inflammatory cascades [29]. In a normal state, NF-κβ units are blocked from activation by IκB kinase. However, under inflammatory stimuli, IκB kinase mediated control is relieved which directs NF-κβ for nuclear import that results in the induction of the inflammatory mediators’ expression. On the contrary, the administration of Curcumin has been found to inhibit NF-κβ activation which is a prime contributor in Curcumin associated anti-inflammatory effects. These effects stem from Curcumin directed inhibitory action on IκB kinase [80]. Hence, other dietary polyphenols having the capacity to modulate this NF-κβ directed inflammatory response can be further dissected to unlock their therapeutic potential in Alzheimer’s disease. Other than this activation of NRF-2, it is also one of the cellular responses seen after Curcumin application, which further acts on the antioxidant response element (ARE) to induce the expression of ARE containing genes such catalase, glutathione peroxidase and superoxide dismutase (SOD) [81]. All of these ARE associated responsive factors help the cells to decrease the oxidative burden of the cell and hence stops the execution of the neurodegenerative program.

**Figure 5:**
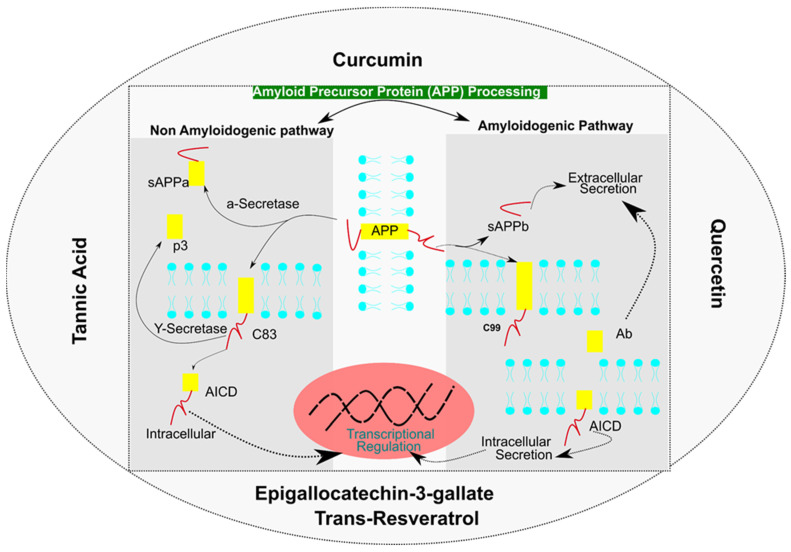
Dietary Polyphenols Influencing Amyloid Precursor Protein (APP) processing by modulating non-amyloidogenic pathway and amyloidogenic pathway.

On the other hand, several dietary polyphenols such as curcumin and quercetin also possess the ability to modulate the Aβ biogenesis pathway. The Amyloid Precursor Protein (APP) is processed through Amyloidogenic and Non-Amyloidogenic pathways. Non-amyloidogenic pathways form sAPPα and p3 fragments, whereas amyloidogenic pathway form sAPPβ and Aβ peptides leading to amyloid plaques. Various other mediators are processed in a sequential manner by α-Secretase, β-Secretase and γ-Secretase to form p3, C83, C99, and an APP Intracellular domain (AICD), which are released into an intracellular or extracellular space. It has been reported that curcumin exhibits the ability to bind to the amyloid β peptide (Aβ) and inhibit or modulate the amyloid precursor protein (APP) metabolism (Figure 5) [82].

Overall, the beneficial effects seen after Curcumin in Alzheimer’s disease pathology seems to be coordinated by its effects on regulating oxidative, anti-inflammatory and Aβ biogenesis pathways in Alzheimer’s disease pathology.

### 7.2. Quercetin: A Flavonoid for Alzheimer’s Disease

Quercetin, a polyphenol categorized under flavonoids, is present in higher plants, fruits and vegetables that are consumed on a daily basis, e.g., onions, apples, berries, asparagus, etc., [68,83]. Their inflammatory and anti-oxidative properties are responsible for most of the effects seen under various conditions [17]. Their anti-oxidative potential is due to the presence of two pharmacophores, i.e., hydroxyl at C-3 positions and catechol which help to neutralize reactive oxygen species (ROS). An indirect effect on AMPK activities also helps to inhibit the NADPH oxidase directed oxidative burden. Other antioxidant effects are contributed by the activation of the NRF2-ARE axis [18,68]. Under conditions such as Alzheimer’s disease, such an antioxidative function coupled with its effect on Aβ production is associated with neuro-protective effects seen under such conditions. Interference in Aβ biogenesis is achieved by influencing amyloid precursor protein (APP) processing through the inhibition of β-Secretase (BACE) activity [84]. Secondly, through the inhibition of NFκβ, quercetin also modulates APP processing (Figure 5) [85]. Other effects seen after quercetin administration is the competitive inhibition of acetylcholinesterase (AChE) and butyrylcholinesterase (BuChE) that helps to increase the level of acetylcholine which is responsible for improved cognitive abilities under mild or moderate Alzheimer’s cases [86]. Owing to the neuro-protective functions seen under quercetin treatment, the only problem that is creating obstacles in its clinical application is the problem of low bioavailability which occurs due to a modification (methylation, sulfation) of quercetin in the gut which decreases its effective concentration [24,87,88]. Although high doses of quercetin can be used to achieve an effective concentration, under in-vitro trials, high dosages have been found to be toxic, whereas anti-oxidative and anti-inflammatory effects have been seen under low dosages [89]. Another concern regarding quercetin’s usage as a neuroprotective agent is its poor blood brain barrier (BBB) penetration properties [90]. The direct administration of quercetin through intravenous and intraperitoneal routes has been used to achieve an effective neuro-protective concentration [19]. Therefore, there is a need to work on the bioavailability concerns regarding quercetin usage in clinical settings.

### 7.3. Tannic Acid: An Aβ Buster

Tannic acid is a plant derived polyphenol (strawberries, apple, barley, coffee, tea, cranberries) belonging to the hydrolysable tannin class which is found to have many health-related benefits [91]. Levels of polymerization in tannin determine their bioavailability and therapeutic efficacy. Structurally, tannic acid is related to EGCG and is expected to have related modes of action. In Alzheimer’s disease, they are found to have an influence on the processing of the Amyloid Precursor Protein (APP) through inhibiting the cleavage on the β-site through their inhibitory action on β-Secretase activity (Figure 5). These events ultimately result in a decrease in the synthesis of Aβ and are found to have an association with improved cognitive function in Alzheimer’s disease [92]. Other intrinsic properties, such as anti-inflammatory, anti-oxidative, anti-carcinogenic and antimicrobial [93], are expected to have additional effects by enhancing its neuro-protective effects. However, the low blood brain penetration of tannic acid demands the development of methods for increasing its bioavailability. The encapsulation of tannic acid in liposomes is one such method which has been found to be effective in increasing the BBB penetration efficiency of tannic acid [94]. Other technologies that can increase tannic acid bioavailability along with a decrease in toxicity can help to increase the effectiveness of dietary polyphenol usage in neurodegenerative diseases.

### 7.4. Mechanistic Involvement of Epigallocatechin-3-Gallate (EGCG) in Alzheimer’s Disease

EGCG is the polyphenolic compound present in green tea. Its remarkable ability to act as an Aβ fibril disruptor makes it an effective compound for usage in Alzheimer’s disease therapeutics [95,96]. Mechanistically, EGCG undergoes ionic interactions with Aβ fiber based on hydrogen bonding as well as non-polar interactions to form an adduct [95,97]. Such an adduct formation reduces the aggregation propensity of these fibers and hence reduces the associated neurodegenerative process seen in Alzheimer’s disease pathology. Adduct formation with other amyloidogenic proteins is also observed [98]. This signifies that the direct effect of EGCG on Aβ kinetics seems to be the major contributor to its therapeutic efficacy against Alzheimer’s disease pathology. Moreover, the EGCG directed modulation of secretase activity reduces the formation of amyloid precursor protein, which subsequently suppresses Aβ aggregation [99]. Additionally, EGCG associated anti-inflammatory effects may stem from its role in suppressing microglia activation which may modulate the course of Alzheimer’s disease [99,100].

### 7.5. Trans-Resveratrol (RV), a Protein Homeostasis Regulator in Alzheimer’s Disease

Protein homeostasis is the prerequisite requirement for the existential need of a cell in the body. Various mechanisms are at play to maintain the proper functioning environment for a protein. One among them is the unfolded protein response (UPR) which gets activated whenever there are defects during the protein folding process in ER and mitochondria. Disordered proteins are subjected to degradation, and the resultant materials are recycled for the next round of the protein synthesis event. However, an abrupt UPR leads to unforeseen consequences. In the case of Alzheimer’s disease, an exaggerated UPR is associated with the progression of the pathology. Dietary polyphenols such as Trans-resveratrol (RV) have been found to attenuate this UPR to influence Alzheimer’s disease pathogenesis [101]. UPR as an important mediator of the RV can be inferred from the requirement of UBL-5 and XBP-1 (the main UPR response associated proteins in mitochondria and ER, respectively) for RV directed downstream effects [102]. A major response seen after RV treatment in the case of Alzheimer’s disease is a 2.5-fold decrease in Aβ plaque deposition [103] which was associated with an effect of RV on increasing the rate of Aβ secretion as well as promoting the ubiquitin proteasome system (UPS), which helps to clear the disordered Aβ and is hence associated with improving the outcome of Alzheimer’s disease [104]. Various molecular events such as the activation of SIRT1 through AMPK contribute majorly to mediating the effect of RV [105]. SIRT1, using its intrinsic de-acetylation activity, mediates the de-acetylation of p-τau protein that helps its clearance through UPS [106]. Hence, the identification of related dietary polyphenols that can influence the protein homeostasis events should be explored in the context of therapeutic strategy development to manage the course of Alzheimer’s disease. According to Capiralla et al., [107] resveratrol also exhibits anti-inflammatory activity against Aβ-evoked microglial activation through a TLR4/NF-κ B/STAT signaling cascade.

## 8. Clinical and Preclinical Aspects of Dietary Polyphenols

For the past 2 decades, tremendous efforts have been made to uncover the clinical efficacy of dietary polyphenols in various diseases. By searching PUBMED with the search term “polyphenols”, 10,718 studies on dietary polyphenols were published between 2001 and 2010, which has grown to 31,452 between 2011 and 2020. This was an approximately 3-fold increase in the interest of researchers in uncovering the role of dietary polyphenols in various disease modalities. So far, in just the last 2 years, 5725 studies have been published. Hence, dietary polyphenol research is an interesting avenue that can be explored for clinical advantage. On the clinical side, as per the previously reported method [108], upon utilizing the https://clinicaltrials.gov/ (accessed on 4 January 2022) search portal for identifying clinical studies related to polyphenols using the search term: Disease: “Alzheimer’s disease” Additional terms: “polyphenols OR flavonoids OR flavanols OR anthocyanidins OR anthocyanins OR isoflavones OR flavones OR flavonols OR flavanones OR flavanonols OR nonflavonoids OR phenolic acids OR stilbenes OR lignans’, to date, there are approximately seven clinical studies. Among them, four studies have been completed and three more are still in the recruitment stage. Flavonoids were the most studied for testing therapeutic efficacy. One study employed soy isoflavone for testing its clinical efficacy. In initial trials, they have found positive effects of Soy Isoflavone on improving the cognitive performance in older adults [109]. Looking at such promising findings, soy isoflavone was further evaluated for its effects on improving Alzheimer’s disease patients’ cognitive performances in older individuals (males and females). Although, this study on Alzheimer’s disease patient did not find any significant effect of Soy Isoflavone on improving Alzheimer’s disease patients’ cognitive performance, but they did find an association of improved verbal fluency and speeded dexterity with an increase in equal (metabolite of soy isoflavone) [109]. Various factors might be associated with the differential response seen as initially expected. The study was done on very old Alzheimer’s disease patients (average age 76.3). Since Alzheimer’s disease is impacted by age, a more extensive investigation including younger age groups might offer additional insight on the therapeutic usefulness of soy isoflavone in the treatment of Alzheimer’s disease pathology. On a similar line, another trial evaluating one of the isoflavones found in soy, i.e., Genistein, was recently tested for its influence on treating Alzheimer’s disease conditions (ClinicalTrials.gov Identifier: NCT01982578). The study was based on the scientific fact that in animal studies, Genistein was able to increase the level of PPARg (peroxisome proliferator activated receptor gamma) which forms dimmers with the Retinoid X receptor to activate apolipoprotein (ApoE) which helps to degrade amyloid beta peptides. Such Alzheimer’s disease attenuating properties of Genistein were expected to be mainly contributing in a decreasing load of amyloid beta protein. Similar effects are expected from the clinical trial that has recently been concluded and the clinical findings from this study are expected sooner. The above two studies signify potential benefits of soy food products in improving cognitive functions and thus need to be further tested in a different geography and ethnicity.

Using functional food is an alternative approach to direct pharmaceutical interventions to deal with Alzheimer’s disease. One such study employing a mixed food supplementation diet has green tea polyphenols, ginsenoside and marine collagen peptides tested for their effects on preventing Alzheimer’s disease pathological signs. This study has just been completed in December, 2019 and results from it are still not available in the public domain (ClinicalTrials.gov Identifier: NCT04279418). Furthermore, various new approaches have been employed recently for conducting clinical trials of polyphenols. Among them, a trial using mail for delivering cocoa flavonols and multivitamins to participants and then evaluating their cognitive performances using a telephonic based questionnaire was explored to study the cardiovascular and cognitive enhancing properties of cocoa flavonols in combination with multivitamin supplementation [110].

In an additional search, so far just using the additional terms mentioned above, there are around 793 studies completed. Most of the studies conducted so far have provided polyphenols in either an extract, a pure compound or a rich food source. Among polyphenols, rich dietary foods (berries, cocoa and dark chocolates, orange, orange juice, cereals, red wine, olive oil, green tea, soy, pomegranates, apples, coffee, potatoes, pulses, beer, hazelnuts, almonds, artichokes and mangos) were of much interest in recent decades of clinical studies. In a pure compound form, flavonols were the most studied, followed by anthocyanidins. Still, there is a large repertoire of dietary polyphenols that can be exploited to gain therapeutic advantages in neurodegenerative diseases.

## 9. Future Directions in Dietary Polyphenol Research for Alzheimer’s Disease

In recent years, much research has been carried out to elucidate the benefits of plant derived polyphenols in treating neuropathological disorders, including Alzheimer’s disease. There is certain evidence that suggests the pathophysiological effects of metabolic syndrome are successfully altered by the dietary intake of polyphenols [20,107,111,112]. For any therapeutic agent to be explored for treating the neurodegenerative disease, it should have the ability to cross the blood brain barrier. Among dietary polyphenols, curcumin [113] and resveratrol [55] have been found to cross the BBB architecture, which makes them ideal candidates for neuroprotective strategy development. Another important consideration for their use is their bioavailability in the body after administration. It has been seen that curcumin reaches the peak limit within 48 h in the brain after oral administration [113]. Other routes of administration, such as intraperitoneal (i.p.), oral gavage and intramuscular, were also successful in increasing the bioavailability of the curcumin [114]. A short shelf life is also another concern regarding the use of curcumin. It has been seen that curcumin undergoes glucuronidation (to form curcumin glucuronides) and modification by sulfates in the intestinal tract and liver milieu which decreases their bioavailability. Other dietary polyphenols, such as resveratrol, etc., also suffer from this sort of bioavailability issue. Therefore, various strategies employing the inhibition of the process of glucuronidation thus will be effective in increasing the concentration of curcumin. Piperine is one such entity present in black peppers (piper nigrum) which is found to be an effective inhibitor of the glucuronidation process. Piperine use in combination with curcumin has been found to be effective to increase the therapeutic bioavailability of curcumin [115]. Other than increasing the bioavailability, toxic response in the form of ulcers, hyperkeratosis, etc., has been seen in the chronic administration of curcumin for a longer duration of 2 years (National Toxicological Program, 1993). Although the chronic therapeutic dosage used in the study is much higher than the level of recommendation, it does highlight the need to examine curcumin’s safe usage within limits. Contrary to this, another strategy that can be used to increase the therapeutic efficacy of curcumin is to make use of combinatorial strategies, e.g., ascorbic acid in combination with curcumin has been found to increase the anti-inflammatory response [116]. Other neuroprotective agents, such as resveratrol and epigallocatechin [117], have also been exploited for this synergistic approach. The positive outcome of such synergistic approaches thus signals the need to expand the horizons and scope of this approach to tackle the menace of Alzheimer’s disease.
